# Embedding violence prevention in existing religious and education systems: initial learning from formative research in the Safe Schools Study in Zimbabwe

**DOI:** 10.1186/s12889-025-23186-1

**Published:** 2025-07-05

**Authors:** Emily Eldred, Ellen Turner, Camilla Fabbri, Amiya Bhatia, Michelle Lokot, Tendai Nhenga, Charles Nherera, Progress Nangati, Ratidzai Moyo, Dorcas Mgugu, Robert Nyakuwa, Sarah Rank, Annah Theresa Nyadombo, Karen Devries

**Affiliations:** 1 Child Protection Research Group, London School of Hygiene & Tropical Medicine, London, UK; 2 Department of Social Policy and Intervention, University of Oxford, Oxford, UK; 3 Academic Research Centre, Harare, Zimbabwe; 4 Childline, Harare, Zimbabwe; 5 Zimbabwe Catholic Bishops Conference, Harare, Zimbabwe; 6 Q Partnership, Harare, Zimbabwe; 7 Formerly Porticus, Nairobi, Kenya

**Keywords:** Formative research, Violence, Corporal punishment, Zimbabwe

## Abstract

**Background:**

Few interventions to reduce violence against children in Catholic Church affiliated schools have been tested for effectiveness. We describe learning from formative research on the development of a school-based behavioural intervention aiming to reduce teacher violence and bullying, which originated from and is embedded within Catholic-run primary schools in Zimbabwe. Specifically, we aim to (1) describe and document the process of intervention development and refinement, including efforts to design the Safe Schools Programme to be embedded into existing religious, child protection, and education structures; (2) reflect on the opportunities and challenges of developing a violence prevention intervention for integration within existing education and religious systems; and (3) discuss the implications for scale-up and sustainability of violence prevention interventions.

**Methods:**

We conducted multi-method research to understand the context of intervention implementation, the acceptability of the intervention, feasibility of the delivery model and to refine both the intervention content and underlying intervention theory of change. This included Theory of Change workshops with all study partners at three time-points, and focus groups, in-depth interviews, participatory workshops at two time-points. Participants in qualitative research included school headteachers, teachers, school staff, priests, students, parents, local government education actors, and child protection NGO staff. Qualitative data were analysed thematically.

**Results:**

Findings reveal several challenges facing schools including low motivation of teachers due to high workload and inadequate school-based referral systems for child protection. Views on the acceptability of corporal punishment are polarised with some parents and teachers supporting its use despite the recent ban, presenting an opportunity for the intervention to support teachers move towards alternative discipline. Findings suggest that aligning intervention activities within existing structures within schools and using familiar teaching methods is an effective way to support intervention uptake while addressing concerns about teachers’ workload and intervention acceptability. The intervention was refined in light of the qualitative findings and Theory of Change workshop reflections, which included: additional behaviour change engagement with teachers, an amendment of the school-based referral system, amendment of manual content for children, and streamlining of materials with existing workload.

**Conclusions:**

Interventions designed by ‘insiders’ at institutions such as the Catholic Church, have huge potential for implementation at a large scale due to systems and context expertise, pre-established relationships, and alignment with stakeholder priorities. However, such interventions should be mindful of power hierarchies and provide adequate support to equip actors with violence prevention expertise. Future research on violence prevention interventions designed by religious institutions and their implications for future scale-up and sustainability is recommended.

## Background

In most countries, children spend a large proportion of their time in educational settings under the care of teachers and school staff [1]. Although most regulation and provision of education across pre-primary, primary, secondary, and tertiary settings are the purview of state governments, religious institutions are major providers of education worldwide, especially in resource-poor settings. For example, estimates suggest that 62.1 million children received schooling from Catholic schools in 2019 [2]. In total, 1.36 billion people are Catholic worldwide, according to the Vatican in 2020 [3]. There is, therefore, a huge opportunity for Catholic schools to positively influence the behaviour of their students and affiliated congregations and communities, in line with Catholic religious belief systems and values [4].

Schools have a key role in creating safe environments for children, supporting their development, and influencing their behaviour and attitudes [1]. Unfortunately, schools can also be sites of violence [5, 6]. Violence against children is not only a human rights violation but a public health issue [7] with well-documented long-term health implications [8–12]. Within schools, children may be exposed to corporal punishment (in which physical force is used with intent to harm or cause discomfort [13]) from teachers and other school staff, violence from their peers (including bullying), and, in some cases, sexual violence. Similarly, due to sexual abuse scandals within the Church, the Vatican is increasing its attention to the role of safeguarding and child protection in the various settings within which it operates [13–15]. A Child Protection Task Force was set up in 2020 to assist Episcopal Conferences, Religious Institutes, and Societies of Apostolic Life in improving guidelines for the protection of minors [16].

Schools and their wider interconnecting structures have huge potential to host interventions that prevent multiple forms of teacher, peer, and other forms of violence. A recent review found 93 evaluations of school-based interventions targeting violence prevention in low-and middle-income countries (LMICs), with the majority focusing on multiple forms of violence, including a combination of corporal punishment, bullying, and sexual violence, (23%) or interventions to prevent bullying between students (23%) [17]. However, an evidence gap map on interventions that address child maltreatment within institutional settings found no studies examining child maltreatment within religious schools or any other religious setting [18].

While this growing evidence on effectiveness of school-based violence prevention programmes is encouraging, most existing interventions which have been evaluated are developed by external actors and thus arguably may be perceived to be ‘outside’ of existing systems [19–22]. Interventions that are designed by ‘insiders’ may be perceived as more in alignment with stakeholder priorities within such systems. However, there are also potential risks, including that insiders could have or be perceived as less expert or credible. Or, especially in child protection, the priorities of the institutions and those who hold power may conflict with aspects of effective intervention practice. Conversely, interventions perceived as ‘internal’ may also face fewer barriers to implementation, especially if run by actors embedded in the same systems. Easy implementation is likely to be of central importance in ensuring sustainability and scalability, but to our knowledge, no such interventions to reduce school violence have yet been evaluated.

The Safe Schools Programme is a school-based violence prevention programme initiated, designed, and implemented by the Catholic Church in Zimbabwe in the primary schools that they manage. The intervention takes a whole-school approach to violence prevention and is designed to be implemented at scale and sustainably.

In this paper, we aim to (1) describe and document the process of intervention development and refinement, including efforts to design the Safe Schools Programme to be embedded into existing religious, child protection, and education structures; (2) reflect on the opportunities and challenges of developing a violence prevention intervention for integration within existing education and religious systems; and (3) discuss the implications for scale-up and sustainability of violence prevention interventions.

## Context

### Child protection, religious, and education sectors in Zimbabwe

In this section, we describe the context in which the Safe Schools Programme has been developed and implemented. A 2018 national study in Zimbabwe found that the lifetime prevalence of physical violence from a parent or guardian for children under 18 was 63.9% for girls and 76% for boys [23]. The study also found that amongst authority figures, teachers were reported as the most frequent perpetrators of physical violence against boys and girls in the two age groups surveyed, 18–24 and 13–17 year olds [23]. Peer violence also takes place frequently within schools, with a national survey reporting that peers were the most common perpetrators of violence against teenage boys in Zimbabwe [24]. In a recent study, Izumi and Rasmussen [25] identified several structural and institutional level factors, that when combined with individual, interpersonal and community level factors, drive violence against children in Zimbabwe, including economic instability and migration.

The Zimbabwe child protection context is undergoing a period of change. The Constitution of Zimbabwe of 2013 makes provisions for child protection in Sect. 81 outlining that all children are equal in the eyes of the law and have a right to be protected from any form of maltreatment and abuse [26]. A new amendment to the Children’s Act was passed in 2023 which aims to strengthen child protection by aligning the Children’s Act with the Constitution and international conventions. Importantly, it introduces new criminal offences against child abuse and requires that professionals working with children in their professional or vocational capacity must report suspected or likely cases of child abuse [27]. In addition, an amendment to the Education Act was enacted in 2020 which prohibited all forms of physical or emotional discipline in schools by teachers under any circumstances [28]. It also envisages alternative forms of pupil discipline, and schools will have to design disciplinary policies in line with regulations to be set and gazetted by the Ministry of Primary and Secondary Education (MoPSE).

Cases of violence and other child welfare concerns in schools should be referred outside of schools to the National Case Management System, which is responsible for responding to abuse and is led by the Ministry of Public Service, Labour and Social Welfare (MoPSLSW) [29]. Within the National Case Management System, Child Care Workers, who are appointed from local communities, and Village Child Protection Committees, identify and refer cases of abuse. However, a 2015 study found that Child Care Workers received relatively little training or support to conduct this work [30]. Further, a recent study found child protection systems are under stress due to corruption, the COVID-19 pandemic, lack of financial investment, qualified professionals especially social workers leaving Zimbabwe, low recruitment of social workers, amongst others [31].

Recent analysis of a Zimbabwe Violence Against Children Survey (VACS), found that 57.3% of children did not know where they could seek help for abuse and of the children that knew where they could seek help 33.1% chose not to and only 9.6% choose to report, with the majority of reports relating to sexual violence [32]. The Protocol on the Multisectoral Management of Sexual Abuse and Violence 2019 and subsequent 2021 Standard Operating Procedure to operationalise the protocol, offer national guidance on how schools can use the National Case Management system.

Within Zimbabwe, lower education sits under the remit of the Ministry of Primary and Secondary Education (MoPSE). Catholic schools are overseen by the Education Commission of the Zimbabwe Catholic Bishops Conference (ZCBC), and managed by a National Education Coordinator, Education Secretaries based in the eight Dioceses, and representatives of religious congregations. Around one-fifth of these schools are owned and run by religious congregations, such as Jesuit, with ZCBC owning and running the remainder of schools. Although the management of these schools are Catholic, staff and students are not required to be Catholic.

In Zimbabwe, schools are undergoing a challenging period, which includes the effects of a two-decade-long economic crisis, with rising inflation that has seen teachers’ salaries decrease dramatically, in addition to the challenges to schooling and staff and students’ livelihoods as a result of the COVID-19 pandemic. These challenges have led to ongoing disputes between teachers’ unions and the government, leading to teachers’ incapacitation and inconsistent capacity to attend schools [33]. Generally, head and deputy head teachers in Catholic schools are employed by and receive their salaries directly from the diocese or religious orders that operate the schools. However, most staff receive their salaries from government subsidies, and the effects of these challenges are relevant to many school staff within these schools.

### Intervention context: role of the Catholic church in education in Zimbabwe

**Fig. 1:**
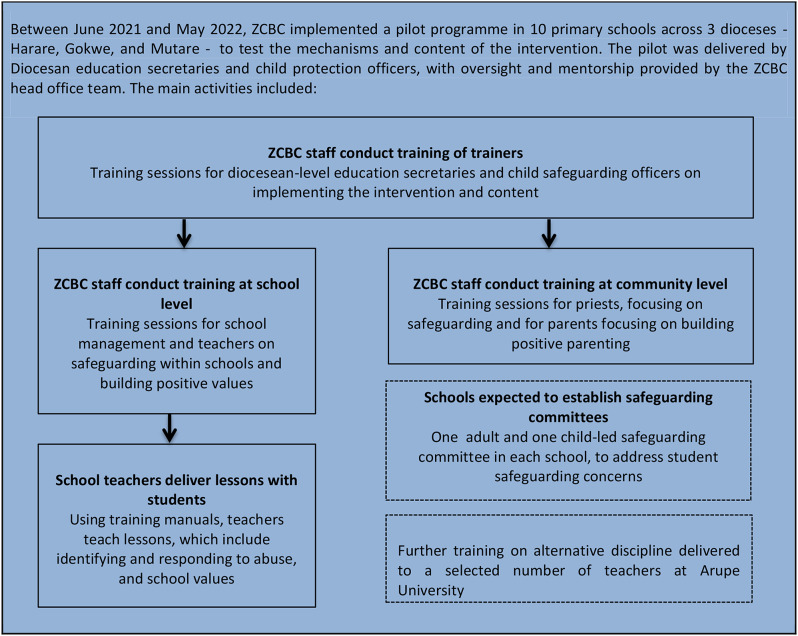
Key Elements of the 2021-2022 Safe Schools Programme Pilot

The Safe Schools Programme is a whole-school intervention designed and led by the Zimbabwe Catholic Bishops Conference (ZCBC) for primary schools managed by the Catholic Church in Zimbabwe. The intervention takes a holistic approach to improving school climate, with intervention content designed to reduce teacher’s use of corporal punishment and prevent peer-to-peer violence in its target year groups. ZCBC’s initial work on child protection began in 2012, leading to the first draft of a national Catholic Child Safeguarding Policy and a national safeguarding sensitisation campaign in 2015 targeted at staff working in the Church. Following feedback from the campaign, ZCBC revised their Child Safeguarding Policy, and focused their efforts on safeguarding in schools. A light-touch school programme was developed and piloted in 2017 in the Diocese of Bulawayo, and after consultation with stakeholders involved in the pilot, ZCBC began to develop a more complex intervention. Between 2021 and 2022, this intervention was piloted in 10 primary schools to test the intervention content and mechanisms (see Fig. 1). We describe further details of the development of the intervention within the Catholic Church elsewhere [4]. Briefly, stakeholders considered priests to hold potential to be key child protection actors in the community, and religious support structures were considered as enablers for diffusion of messaging within the religious congregation. However, research also indicated challenges including the need to establish functional referral structures that hold perpetrators of abuse accountable, including religious actors.

## Methods

### Study design

In 2019, the Safe Schools Study was established to support the development and refinement of the Safe Schools Programme, and to evaluate its effectiveness. The research is a collaboration between the London School of Hygiene & Tropical Medicine (LSHTM) with expertise in formative research and evaluation, the Academic Research Centre (ARC) bringing expertise on qualitative research and child protection, Q Partnership (Q) managing the qualitative and quantitative data collection and M&E, Childline Zimbabwe (CLZ) as the child protection partner and Porticus who is the funder and leads on programme management.

**Fig. 2:**
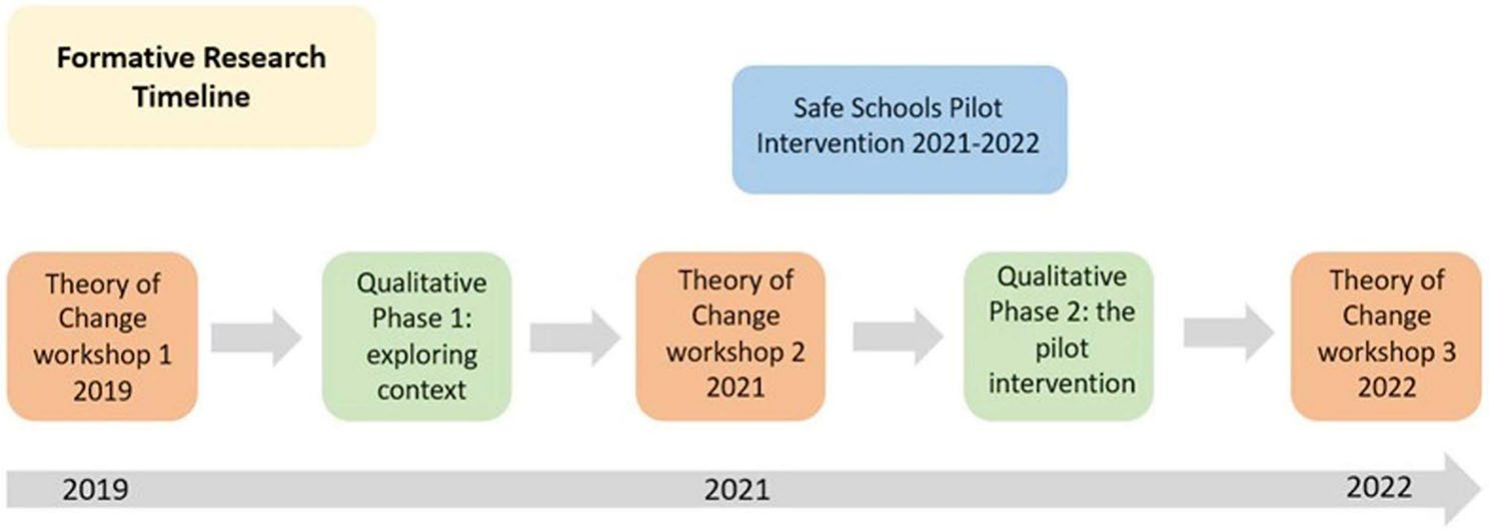
Safe schools study research process and intervention refinement timeline between 2019–2022

In the Safe Schools Study, we follow MRC guidance on evaluating complex interventions, which recommends a phased approach to exploring theory, intervention mechanisms, and trialling the intervention [34, 35]. As part of our initial work to develop a programme theory, understand intended intervention mechanisms, and acceptability and feasibility of implementation, we conducted a Theory of Change process; qualitative research to understand the context; qualitative research alongside a pilot test of the intervention to examine feasibility and acceptability; and further refined the Theory of Change. The overarching research questions for the formative qualitative research included: What are the most significant forms of violence that children experience in schools and communities and how are these understood and referred to by key school stakeholders (pupils, teachers, school management, and parents)? What kind of interventions do key school stakeholders think would be needed to address these forms of violence or have already been effective? How do key school stakeholders think these interventions would be received, and what do they see as the key barriers and facilitators for successful implementation? Fig. 2 outlines the timeline of the formative research alongside the intervention development stage.

In this paper we present data from the Theory of Change (ToC) workshops and qualitative research. We applied an iterative process, whereby research findings from each activity informed the next activity. For instance, the specific research questions and refinements to design for the qualitative phases were informed by the ToC workshops, and/or the previous qualitative work. In addition, these activities highlighted areas for refinement to the research.

### Theory of Change workshops

The Theory of Change (ToC) of a programme or intervention articulates the causal connections between the activities and outcomes and it is intended to offer a description of how activities produce a series of results that contribute to achieving impact [36, 37]. The ToC of the Safe Schools Programme was developed through an iterative process over a 4-year period.

ToC workshop 1 was held in-person over two days in Harare in July 2019, with the following objectives: (1) to formulate a clear impact statement and identify key components of the intervention; (2) to recognise and reflect on the legal, institutional, and cultural context in which an intervention would take place, including the potential barriers and facilitators to change. The meeting involved participation from the ZCBC team, education representatives, such as teachers and headteachers, Catholic actors at a range of levels (Bishops, Education Secretaries, Jesuit stakeholders with extensive experience in violence prevention interventions), NGO representatives with experience in child protection and safeguarding, and the Safe Schools Study team.

A second workshop, held online due to COVID-19 related travel restrictions, took place in February 2021 (ToC workshop 2) to convene members of the research consortium. The team participated in several presentations and group discussions to share findings from qualitative research conducted in October 2020 - January 2021 and to describe assumptions underlying the intervention activities that would be tested during the pilot implementation of the intervention. The team also drafted an initial diagram to map out the key ToC elements (activities, outputs, and outcomes) along the hypothesised pathways of the intervention.

In July 2022, a third in-person workshop was held over three days (ToC workshop 3), followed by an external stakeholder engagement meeting. The third ToC workshop aimed to engage partners to reflect on the pilot implementation of the intervention which had taken place from 2021 to 2022 and to share findings of qualitative research conducted in March 2022: to identify what had worked well and what would need to be refined, and streamline intervention outcomes and associated causal pathways. Participatory approaches and group-work were used to identify outcomes of interest and outline the associated outputs generated by the intervention activities. The team collaboratively updated the ToC diagram which was shared and validated with external stakeholders such as representatives of NGOs and religious bodies as well as government officials. All meetings and workshops were facilitated by researchers from the London School of Hygiene & Tropical Medicine, in collaboration with Q, ZCBC, and ARC.

In May 2023, following further revisions to the intervention and discussions with partners, the ToC was refined further.

### Qualitative research

Research (conducted in October 2020-January 2021) was designed to explore: (1) how different forms of violence against children are understood and referred to by key school stakeholders; (2) what school stakeholders think is needed to address violence; and (3) how school stakeholders think such an intervention would be received, and their views on barriers and facilitators for effective implementation. Interview topic guides covered topics such as understanding of child protection and safeguarding; forms of abuse children face; areas of importance for a safeguarding intervention; views on corporal punishment and alternative discipline; and some additional questions according to area of expertise.

Qualitative research conducted in March 2022 towards the end of the ZCBC pilot implementation period aimed to understand how the pilot intervention Fig. 1) was received by school staff, students and stakeholders, and to identify barriers and facilitators for wider implementation and for intervention effectiveness. We also aimed to understand the context of children’s experiences of violence and disclosure of violence in their schools. Topic guides covered issues such as challenges for children in school and their response to these, experiences of violence, barriers and facilitators to the intervention, perceptions of the intervention, suggested changes to the intervention, and linkages between the intervention and local actors. Table 1 outlines further details of each phase of the qualitative research.

**Table 1:** Research participants and study setting

	Participants	Research Setting	Additional considerations
Phase 1	Key informant interviews (*N* = 18) were conducted with: 4 parents (2f/2m); 4 teachers (2f/2m); 2 school priests (2 m); 2 headteachers (2f); 6 higher-level stakeholders external to the schools, including local government education actors (2 m), education actors within the Catholic Church (2f/1m), and one child protection NGO actor (1 m).	1 school in a high-density, urban setting in Harare and 1 school in a rural setting.	We postponed planned engagement with pupils due to safety concerns amid school closures and COVID-19 lockdowns, and therefore were not able to engage school children in this study.
Phase 2	Sex-differentiated participatory group workshops with students (*N* = 4); individual critical incident interviews (*N* = 4); focus group discussions (*N* = 6) with students (aged 8–12), teachers, parents who were involved in the intervention activities; individual interviews with headteachers and priests (*N* = 4); school-wide qualitative observation forms (*N* = 2); individual interviews with key stakeholders external to the school (*N* = 6), including local government education actors, key ZCBC education actors; and child protection workers in the local area, and focus group discussions (*N* = 2) with key local child protection actors.	Data was collected in two of ten Safe Schools pilot intervention schools towards the end of the one-year pilot of the intervention.	Schools were selected for their participation in the pilot implementation, for their location within the Archdiocese of Harare and daily driving distance of Harare, and for one urban and one rural setting. In both schools the pilot intervention was almost complete, however, the component on referral mechanisms was not part of this pilot trial.

### Qualitative research procedures

For both studies, study sites were selected in collaboration with ZCBC and approach for participation was initiated by ZCBC. ZCBC identified high-level stakeholders, including local government education actors, relating to their experience of child protection and potential role in the intervention. We chose schools to contrast between rural and urban settings, that were within one day’s driving distance from Harare for safe accessibility during the COVID-19 pandemic, and where staff spoke standard Shona. School headteachers consented to school participation, and supported the research team in selecting teachers and parents who were willing and able to participate in the research. All participants were offered the choice to conduct the interview in Shona or English, with many interviews and focus groups combining both languages. This enabled participants to converse in the language that they were most comfortable with. Although there was potential for issues with equivalency with some words, the tools were reviewed by co-authors who were multi-lingual in English and Shona to ensure accuracy. Data collection was conducted by qualitative researchers who had experience in this context, and who had undergone training on conducting research into violence against children. Social welfare officers accompanied the field researchers to handle any emotional issues or stressful situations that could arise needing immediate professional attention and referral process, particularly in research activities involving children.

### Qualitative data analysis

All data was transcribed, and translated into English where necessary, following data collection by the qualitative research team and checked by senior members of the research team for accuracy. Where transcripts were translated to English, 10% were back-translated to Shona by Q researchers to ensure accuracy. All data were inputted into NVivo data management software for coding and analysis. Analysis of qualitative data for phase 1 was led by LSHTM (ET) in collaboration with Q (RN) and ARC (TC and CN), while for phase 2 it was led by ARC (TC and CN) in collaboration with Q (RN) and LSHTM (ET). The data analysis approach for both studies was thematic, with key themes identified that were relevant to the aims of each of the two phases. For coding we drew on both an inductive and deductive approach which involved several rounds of coding and reorganising themes, as follows: an initial code list was drafted prior to the research drawing on the literature and based on discussions with the wider team, including lead education practitioners, international academic researchers in the fields of education and public health, and academic researchers with expertise in law and education in the Zimbabwean context. Subsequently, the qualitative research teams (led by senior qualitative researchers) in each respective phase coded the data using the constant comparison approach [38] to identify codes emerging from the data. Finally, the key themes that emerged out of these two coding approaches, were shared with the broader research team. These themes were discussed with the broader research team until consensus was achieved. Data was coded using NVivo alongside theme identification in an iterative process, to ensure that themes were both relevant across the data and were responding to key emerging issues in the data. Emergent themes were shared with the broader study and ZCBC implementation teams as part of the Theory of Change processes (Theory of Change workshops 1 & 2), with the final themes and important issues being finalised following the relevant Theory of Change workshop for each study.

### Ethical practice and procedures

Ethical approval was obtained from the London School of Hygiene & Tropical Medicine (LSHTM) and the Medical Research Council of Zimbabwe (MRCZ) [MRCZ/A/2552]. All researchers underwent training in conducting sensitive qualitative research into violence against children, and handling disclosures of abuse and participant distress. Written informed consent was obtained from all the adult participants. For adults, interviews were conducted in private locations where participants could not be overhead, and participants were reassured that their data would remain anonymous except if they disclosed that they had hurt a child. Focus groups followed similar procedures but obviously anonymity could not be guaranteed. For research with children (phase 2 only), children provided written assent and written informed consent was obtained from their respective guardians. Research activities for children took place where they could be seen, but not overheard. Children were reassured their data would remain anonymous, except if they disclosed that someone had hurt them and they needed further support. For both consent and assent procedures, information sheets and consent/assent forms were read out to participants with opportunity for clarifications and were then recorded in written format by the researcher. Adult participants had a printed copy of the information sheet which they could refer to while these were explained by the researcher. For the small number of remote interviews conducted as part of phase 1, we describe our methods in more detail elsewhere [39]. Beneficence was explained as having the opportunity to contribute to abuse prevention and/or better schools in participant communities. A referral protocol was in place to handle and refer disclosures of violence against children, in collaboration with study partner Childline Zimbabwe. This included levels of severity of disclosure, and associated referral timeframes and actions.

## Results

In this section we reflect on learning from the three ToC workshops and the qualitative research. We present these results chronologically. Figure 2 outlines the timeline of the two phases of qualitative work and the three ToC workshops alongside the intervention. In the qualitative work we explore key themes relating to the existing school, corporal punishment and child protection systems: socio-political context in and around schools; intervention delivery approach; opportunities to align with existing activities; engagement with external actors; the context of corporal punishment and alternative discipline; the importance of effective alternative discipline; existing referral mechanisms for violence; aligning with schools systems; understanding context; and embedding within child protection systems. We then explore implications for the current intervention design.

### Theory of Change workshop 1 (2019)

The initial ToC workshop proceeded any qualitative data collection and participants formulated an impact statement for the intervention: “an overall school environment where teachers and students would feel free and safe based on a culture of safety, mutual respect and love” to be achieved through reductions of violence from school staff, reductions in peer-to-peer violence and improved school climate. The workshop led to the identification of key intervention components such as a school safeguarding policy, school scorecards to create action plans and monitor progress within schools, life skills training targeted at teachers and students to promote positive relationships, as well as outreach to the community through existing school structures.

### Phase 1 qualitative research: Understanding intervention context (2020–2021)

#### Context-responsive: socio-political context in and around schools

Schools in Zimbabwe are currently undergoing a highly challenging period, posing challenges for the intervention. Participants discussed the huge challenges that school staff faced, leading to low levels of motivation, and emotional and practical challenges for staff, which would impact their response to the intervention activities, as shown in Table 2. Teachers’ perceptions of, and motivation for, engaging in child protection activities were shaped by these challenging conditions [40]. These findings suggest that the intervention will need to be seen as supportive for teachers, and take account of these challenges; limit additional work for teachers; and ensure ongoing follow-up and support over time.

**Table 2:** Phase 1 qualitative research: context

Intervention delivery approach is well-suited to context	[Child protection policies] are very necessary, essential and crucial. We can’t do without them because for a good operation we need policy, we need guidelines, yes love might be natural but we need systems and guidelines to do that School priest, rural area
	Are we equipped, are we ready, do we have the resources, are we trained, have we gone through workshops, are the parents informed, is the child informed? All the stakeholders, are they trained on how to live in the new normal? Headteacher, urban school
Socio-political context in and around schools	[Teachers face] emotional abuse, in the event that the teachers are incapacitated like in our community, the parents then when we try to go home, you can hear them selling their things saying ‘the teachers time is up’, meaning to say the money that I have worked for in the day is nowhere near what they make in a month. So, in a way you will be emotionally abused Female teacher, urban school
	I mentioned the issue of teachers who are distraught by their salaries and much of our energy goes there. We have many conditions to meet, conditions that may not be in line with child safeguarding School priest, urban area
	It’s also true that teachers are disgruntled, the moment you say child protection is an extra responsibility then it becomes an issue Catholic stakeholder 1
	…whatever programmes you may take to the schools they may say ooh this is good, but the moment you leave the school, and you don’t make serious follow ups you will be surprised and shocked…that remains an issue that the schools they need to be monitored constantly, consistently Catholic stakeholder 2
Context of corporal punishment and alternative discipline	There is the issue where corporal punishment is not allowed but [we should use] other alternative disciplinary action. For example, I have a teacher who would take two students that were fighting, give them an exam and tell them that they had to score a minimum of 60%. I feel that method takes long in reforming a child. If you really want a child to learn then corporal punishment is the best. Whipping has instant justice Headteacher, rural school
	Corporal punishment is not the best way of disciplining children. You will be harming them physically and once they get used to caning it will be difficult to control them. I believe and understand that there are other ways of disciplining children, like always keeping them busy Male teacher, urban area
	There are parents who are saying beat my kid and there are parents who are the opposite. I think we are at a point where our culture is shifting and it is a stage where you also find resistance and buy-in. So it is still a mixture of ideas and points of view. This is not a reason to stop; it is the reason to educate people School priest, urban area
Existing referral mechanisms for violence	If you come across some cases you will not take them further, because you know that if the case proceeds, justice will not prevail. So, these are the things that other people do not consider, of course we talk to the children and try to empower them, but when you get into the case and try to get justice you won’t prevail Female teacher, urban school

#### Intervention delivery approach within existing systems

Qualitative findings suggested that the intended intervention delivery approach was well suited to its context, and that this may be a strong facilitator for the intervention. The Catholic Church and Catholic primary schools were perceived by participants as well positioned to deliver the intervention and promote its messages more widely in the community [4]. Secondly, the approach and strategies used by the intervention align well with familiar approaches used in Zimbabwean schools, and that participants felt were important to create change around child protection practices. These included: a clear overarching policy, workshops for school staff and parents, and resources such as manuals, posters and handouts to raise awareness and garner support. However, the familiarity of these structures and approaches could mean that intervention messages could become diffuse, or may overlap with existing school structures. This suggests that the intervention will need to have clearly defined boundaries.

#### Context of corporal punishment and alternative discipline

Corporal punishment was seen by many parents and school staff as being a form of discipline that was quick, effective, and could be essential to punish and prevent more serious misbehaviours. These respondents also indicated that corporal punishment was used in their own upbringing and they did not therefore see anything wrong with it as they, “turned out fine”. The study also revealed that the teachers had a narrow understanding of corporal punishment, limited to “beating” but not including other forms of punishment such as painful tasks. School staff tended to express some interest in, but also ambivalence towards, alternative discipline approaches. In addition, as we explore elsewhere [40], teachers perceived corporal punishment to be routine and moderate and not a form of violence against children. However, some school staff held the opposite view and described being open to alternative discipline approaches. Participants reflected that perceptions around corporal punishment and discipline strategies were undergoing a period of change in Zimbabwe, as shown in Table 2.

These findings suggest that this is a strategic moment to implement this intervention. However, at the same time, strong beliefs uphold the use of corporal punishment in schools, and school staff have concerns about the effectiveness of alternative discipline approaches. This suggests that the intervention will need to both engage in a meaningful process of change around social norms supporting corporal punishment and also offer school staff strategies, training, and support on alternative discipline approaches.

#### Existing referral mechanisms for violence

The findings also suggested that the current referral mechanisms for violence in schools face some challenges, including cases of abuse that were handled poorly that left individual staff feeling isolated in handling the case and did not lead to positive outcomes for the child involved. As shown in the quotations in Table 2, this includes practical challenges a teacher experienced in supporting a student through the legal process [41], suggesting positive outcomes for children who report cases are challenging to achieve.

### Theory of Change workshop 2 (2021)

During the 2021 workshop, findings from the phase 1 qualitative research were discussed. Several key reflections emerged: school actors are in school despite socio-economic challenges and are able and willing to engage with intervention activities; scorecards that allow actors to monitor and assess the status of the school environment promote the creation of effective action plans for school improvement; schools set up internal safeguarding committees to promote awareness and engage the school community; referral mechanisms can be established within schools; and linkages with formal systems can be achieved. The achievement of intervention outcomes was based on three main conceptual hypotheses: teachers’ upskilling and training in alternative discipline would lead to the replacement of corporal punishment in favour of positive discipline; effective referral structures and sanctions for perpetrators would contribute to lowering levels of violence within the school; awareness raising and shifts in social norms and acceptability of violence would complement and sustain reductions in violence and would improve relationships between school actors.

Some key considerations that emerged from group discussions, in line with several qualitative findings from phase 1, were: (1) teachers’ existing workload is high and motivation levels in some areas are low therefore intervention activities should try to be responsive to teachers’ needs and build on existing activities; (2) the need for the school-based referral structures targeted through the intervention to be effectively integrated into the national case management system [29] with clear guidelines to ensure the safety of those reporting/disclosing any form of violence; (3) behavioural change requires capabilities, opportunities and motivations [42] in addition to awareness. This feedback was used to make changes to the pilot intervention that was implemented in 2021–2022 in 10 schools across 3 dioceses.

### Phase 2 qualitative research: Understanding acceptability of intervention and feasibility (2022)

In phase 2, through research alongside the pilot intervention, we identified five further key elements to consider in further revising the Theory of Change, as discussed below.

#### Opportunities to provide effective alternative discipline approaches

The findings suggested that school staff largely appreciated the approach taken by ZCBC to support them in implementing the recent ban on corporal punishment, which had been identified as a concern in phase 1. However, adults also expressed trepidation about the corporal punishment prohibition, including uncertainty about the effectiveness of alternative approaches. Similarly to our findings in phase 1, some stakeholders upheld beliefs supporting corporal punishment, and perceived that children would misbehave without it, as shown in Table 3. In addition, some parents expressed concern about teachers failing to actively manage students’ behaviour.

**Table 3:** Phase 2 qualitative research on acceptability and feasibility

Corporal punishment and alternative discipline strategies	I just wanted to say that we all know in here that we used to be beaten by our teachers and it helped us to know what was required of us at school but this new law has led to children abusing the parents and the teachers. Children no longer listen because they are no longer being given corporal punishment Community care worker
	Teachers must be educated that if the law says children must not be beaten up it does not mean that they must now neglect or leave the children doing whatever they want […] Some teachers will just sit and watch the children because the law said they must not beat them up Parent, urban area
	I think there is need for some awareness to everyone that instead of beating up children we can use other means […] It requires a teacher to know how they can best treat a child when they do something wrong, they need to know the ways that will make a child become a good person and the parents too must know the ways that will lead to the development of their child into becoming a good person Parent, urban area
	The problem I have with our Ministry is that they just give out laws without training or even explaining the law. If it wasn’t for the Archdiocese that came to explain and train us, we would still be using the old ways […] Because of the Archdiocese training we now speak with one voice. I was empowered and equipped on how to handle children without using beatings and how to explore other methods. It also helped in managing our own anger issues. Teacher, rural area
	We thought that raising my voice was the only way to be heard but now we know that good communication is the best. So child safeguarding training has helped a lot. It has even helped in the marketing of the school because children now love coming to this school because of how we interact with the children. Teacher
Opportunities to align with existing activities and structures	We have had problems with some teachers at school level complaining that because of the new curriculum this is an additional load […] That is our worry. We have a syllabus, in that syllabus we have Guidance and Counselling, they include child safeguarding and protection issues then it should be integrated in the school system, then there is no problem Headteacher
Aligning with school systems: Manuals and content	It’s like the topic starts with instructions on what is supposed to happen in the lesson. I don’t have to necessarily agree with the instruction because I’m the one on the ground and what the instructions says it not relating to the children in the class […] I have to think of way to make sure the children understand. I take the activities in the learner’s book and add my own flesh to it. I tried to follow the teachers resource book and I found it complicated my lessons that’s why I now stick to the learner’s book Teacher
	You see an image of a child being burnt using matches on his head, that I don’t like…. Most of the stories relate with children saying the child did this and this yet it’s so sad and pitiful to see a child being abused Learner, rural area
	I don’t like this book because it has few pictures of things, for example at this school others can’t read, so if they use pictures, we can then explain for them to understand through the use of pictures Learner

Adult stakeholders described being open to using alternative discipline strategies, provided there were suitable alternatives that were effective to manage children’s behaviour. Some participants in particular expressed feeling supported by the ZCBC intervention, and appreciated the guidance it gave that they felt would help to protect them in light of the changes to the law. One teacher explained, ‘this program… is really good because if I beat a child that will be the end of my career as a teacher’, while another described feeling ‘empowered and equipped’ to handle children’s behaviour without beating, as shown in Table 3. This contrasted with policy approaches which were seen as punitive and unsupportive.

#### Opportunities to align with existing activities and structures

Findings from phase 2, building on the similar findings in phase 1 and the TOC workshop 2, suggested that there was some overlap and opportunities to align the ZCBC intervention activities with existing activities already ongoing in the school. This included some overlap between, for example, the work of the existing School Development Committee and the intervention adult-led child safeguarding committee, or the aims of the existing 'Guidance and Counselling' curriculum and the intervention learners’ manual. This had positive implications, as some teachers felt that this added motivation for the activities, as it could support them with this other ongoing work, however duplication of effort was also recognised. This suggests that there is opportunity to align intervention activities with those already underway in the schools, and this may provide further support and motivation for teachers.

#### Aligning with school systems: manuals and content

Manuals had been identified as a familiar approach and potential facilitator of the intervention in phase 1 and were tested in the pilot intervention. During the pilot intervention, teachers and learners expressed mixed responses to the manuals, which suggested some variability in how they were both implemented and perceived. First, the findings suggested that teachers may be implementing and teaching the manuals in varied ways. While learners in one school felt that the intervention focused on ‘good manners’ and children’s behaviour, leaners in the other school emphasised the intervention focus on abuse against children, as shown in Table 3. Some teachers themselves also described adapting the intervention content to be suited to their classes, and suggested differences between suitability for urban and rural settings - which may have implications for intervention aims. Further, some teachers also discussed that the learners’ manual was more helpful than the teachers’ manual, as it had more detail and was easier to use. Refining the manual to offer clear and constructive guidance for teachers may be important.

Second, learners also described different experiences with the manuals. While some children found the intervention content clear and easy to understand, others found it confusing and stated that more pictures and easy-to-read language was important. Some children also felt upset by the content on abuse. This suggested that overall, the learners’ manual would be strengthened by more images and child-friendly language to ensure readability and acceptability for all children, and that left children feeling positive.

#### Opportunities to guide referral mechanisms for cases of abuse

The findings from this phase further reiterated the observations emerging in the previous phase of research, suggesting that referral mechanisms for reporting cases of abuse will face some challenges. In particular, while some children felt comfortable reporting their experiences to teachers, peers or the police, some others felt highly mistrustful. A number of children, particularly female learners, discussed feeling fearful that if they were to report experiences of abuse within intervention schools structures, such as child-led committees, they may be laughed at or not taken seriously by their peers or even by their teachers. Further, some girls also described fearing the police themselves, through not being believed or even experiencing abuse from the police while school staff feared repercussions from perpetrators. This also poses significant challenges regarding ensuring children’s cases are handled safely, respectfully and confidentially by actors outside the school, and that those referring cases are supported, as this is outside the remit of the intervention. This suggests that encouraging children to report to mechanisms and actors outside the intervention itself, should be very carefully considered.

At the same time, however, school staff and learners also appreciated the guidance and support offered to them on recognising and responding to abuse, and children enjoyed being active in issues relating to their own protection. This suggests there are opportunities to incorporate this positive engagement with school staff and learners across other aspects of the intervention, and that this could be separated from the referral mechanisms themselves.

#### Engagement with actors external to the schools

The findings suggested that buy-in and support from key actors in the community, external to the schools, will be important for its success. This included actors at a range of levels, including community leaders, particularly in the rural area, and government actors at a national and district level. The need for a government-down approach to motivate all involved to support the intervention, was emphasised. However some participants described challenges, particularly in rural areas, suggesting others such as community leaders and Church structures may need to be engaged.

### Theory of Change 3 (2022)

During the third ToC workshop in 2022, the relationship between the Catholic Church and local government structures and stakeholders was discussed in light of phase 2 and identified as a key factor influencing how the intervention was received, highlighting the need for consistent engagement of local actors across implementation areas (dioceses). Intervention activities were positively welcomed by school actors in the context of the recent corporal punishment ban, however, concerns were raised around teachers’ ability and willingness to use alternative disciplinary methods, especially amidst concern from parents. Additionally, some capacity-building activities and structures promoted by the intervention were perceived as duplicating and occasionally conflicting with existing school structures. Suggestions for improving the intervention materials, following discussion on some difficulties with the manuals that emerged from phase 2, were offered by participants to make the content more user-friendly and calls for sustained mentoring, coaching and facilitation for schools were made by intervention coordinators. Teachers’ already high workload, coupled with limited training on the complex intervention content, were seen as key barriers to intervention effectiveness. Contextual factors, consistent with the qualitative findings, related to teacher demotivation and challenging economic conditions were also mentioned as important influencing factors.

The intervention was refined between 2022 and 2023 following reflections from the three ToC workshops and qualitative findings. A subsequent theory of change discussion with all partners took place in May 2023, leading to the current iteration of the ToC.

### How did the qualitative study findings and ToC workshops feed into intervention refinements?

In light of the qualitative and ToC workshop feedback, several key recommendations for intervention revision emerged. Firstly, stakeholders noted that many of the pilot intervention activities were hugely successful in increasing awareness of safeguarding and prevention of corporal punishment in schools among students and school staff. However, in order to create substantial and sustainable behaviour change, the intervention will require ongoing and repeated engagement in activities across a long period of time. The intervention refinements, include: regular 30-minute sessions with teachers on alternative discipline practises; and ongoing support by the ZCBC team for teachers to reflect on learning.

During the pilot implementation, the intervention components aimed at strengthening referral structures within schools were still being designed and therefore they were not tested in this phase. The pilot experience however sufficiently illustrated the challenge of embedding intervention activities within the already complex school structures. The intervention team therefore decided to amend this component of the intervention because the expertise, resources, and time required to design and implement safe and effective referral systems was considered too high to be achieved within the scope of the intervention.

The need for more user-friendly documents and manuals emerged as a key priority for intervention refinement from the qualitative work and the ToC workshop. The intervention refinements therefore include using more child-friendly images in materials, reducing the volume of text and making the content accessible for non-Shona speaking learners. Findings highlighted that there was a need to refocus the content on the three key outcome areas, identified in the theory of change workshop, and include examples of behaviour changes that exemplified these practices.

Understanding and attending to the wider context in school and the community was highlighted as a key priority for the intervention refinement. As noted in the qualitative findings, demotivation of teachers was a wider concern in primary schools across the diocese. The intervention refinements will streamline and simplify the materials so as not to cross over with existing curriculum materials and thus overburden the teachers. Opportunities to align content with the existing ‘Guidance and Counselling' curriculum were discussed as a possibility. In addition, school staff may experience both personal challenges and possible experiences of trauma. Thus, the intervention will be framed around positivity, for example framing the communication around ‘positive’ parenting and teaching.

**Fig. 3:**
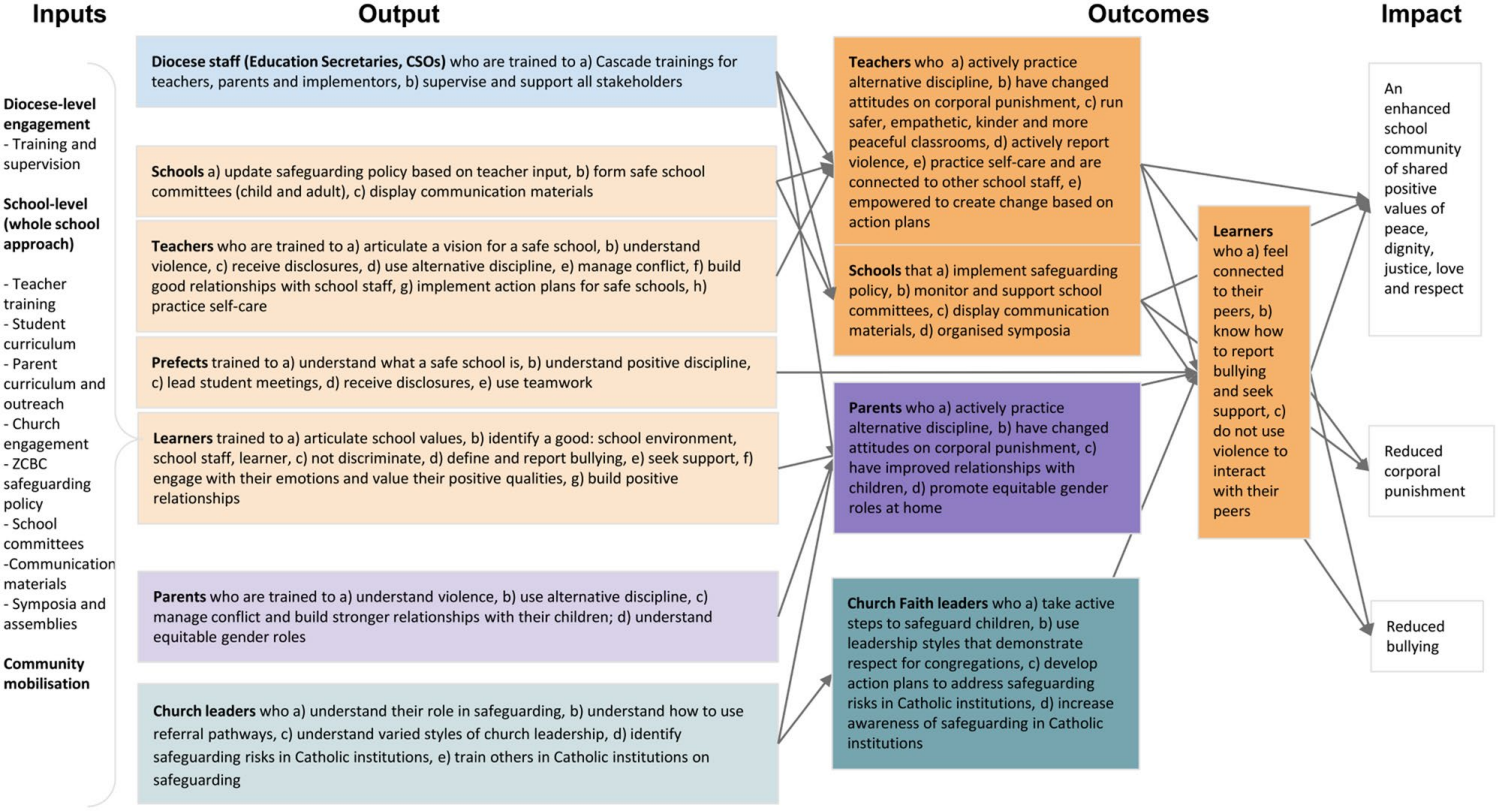
Current iteration of the Theory of Change Model

The ToC workshops and reflections from the qualitative findings, coupled with the intervention refinement process in 2022–2023, led to the identification of three main long-term outcomes that would contribute to achieving the overall intervention goal of creating “a school that is a place of safety, belonging and happiness for all where children’s whole development is supported through the mutual respect, collaboration and accountability of teachers, parents, community and children, based on positive values”. The main outcomes of the intervention are: (i) reduced corporal punishment, (ii) reduced bullying, and (iii) an enhanced school community of shared positive values of peace, dignity, justice, love and respect. Figure 3 outlines the current iteration of the model, including the refined intervention inputs, outputs, outcomes and impact.

#### Reduced teacher violence

Improving knowledge and practice of positive discipline among teachers and prefects, through regular training, would promote the replacement of violence in favour of alternative methods when accompanied by awareness raising activities aimed at shifting norms alongside behaviours. Collaborative teaching methods that involve students in decision-making and peer mentoring and coaching between teachers would lead to improvements in teachers’ empathy, self-confidence, and self-efficacy and consequently to better student-teacher relationships. Better relationships coupled with self-care and empowerment to create change and disciplinary practices would contribute to lower levels of violence from teachers.

#### Reduced bullying

At the same time, strengthening students’ self-confidence and communication skills would also promote better relationships between school actors and would help students exercise self-discipline. Students’ engagement in collaborative activities in class and at home and opportunities to practice positive behaviours would not only strengthen positive relationships but also increase students’ feelings of connectedness to the school environment and benefit from gains in self-confidence and pride. These changes in students skills and behaviours will lead to lower levels of bullying.

#### An enhanced school community of shared positive values

Activities aimed at engaging school management, teachers, parents, and students towards building a common understanding of the school values and rules would generate feelings of collective ownership of safeguarding within the school. Value-based reflections and initiatives that bring together children and adults to promote safeguarding and respect would further strengthen relationships and bonding within the school and build strong support systems leading to positive school climate and culture.

## Discussion

In this paper, we describe and document the process of intervention development and refinement, including efforts to embed the Safe Schools Programme into existing religious, child protection, and education structures. We find several challenges facing schools including high workload for teaching staff and inadequate school-based referral structures for cases of abuse. In addition, we find divided views on the use of corporal punishment at schools from teachers and parents, with many supporting its use despite the recent ban. Our findings suggest there is an opportunity for the intervention to play an important role in supporting teachers to move towards alternative discipline strategies, through recognisable structures and teaching methods, such as manuals and school committees. Findings suggest these familiar methods of teaching will not only serve to support already overburdened staff but will also help integrate the intervention within the existing school structure. ZCBC’s systems expertise and pre-established relationships with the Church, schools and ministries (MoPSE) will be critical to developing this familiar approach and in easing the intervention acceptability amongst these stakeholders, which our findings suggest will play an important role.

However, our findings suggest that additional training may be needed to equip intervention actors and existing change agents within schools with appropriate content, ethical and safety skills relevant to violence prevention. For instance, findings highlighted the need for intervention content to be more child-friendly, and to not perpetuate harmful ideas, such as blaming children for abuse, as explored elsewhere [41]. In addition, qualitative findings, alongside prior literature [30, 31], highlighted the challenges with the wider referral system in Zimbabwe and the difficulties of integrating this with a school-based referral system. The current mechanisms within schools faced challenges, including poorly handled cases and negative outcomes. Although strengthening the referral mechanisms was part of the initial intervention design, these were not piloted. The current challenges, alongside the limited content expertise, highlighted the need for this aspect of the intervention to be carefully considered in the intervention refinements.

Given the current challenges within and around schools explored in this paper, the findings suggest the intervention will need to align closely with existing school, child protection and religious systems to be successful. However, the ToC workshop discussions highlighted the need for the intervention to have clear boundaries around its intended content, or intervention messages could become diffuse, and may overlap with existing structures in the schools. Balancing between aligning the intervention within existing systems and setting clear boundaries around it will also be essential to successful delivery and acceptability by intervention beneficiaries and stakeholders.

### Comparison to other literature

Relatively few studies document how violence prevention interventions in schools are conceptualised, designed, and developed, and along with previous work [4], we provide learning for future formative research in preparation for evaluation and impact analysis. In violence prevention, interventions are often designed by, or in consultation with, behaviour change or topic specialists, NGOs, or academics [19–22]. These interventions are then tested in randomised controlled trials (RCTs) which are often carefully controlled, documented, and delivered by well-resourced and/or specialised organisations. Few interventions evaluated in RCTs have been designed from within national or local education authorities, yet, contextually-informed, well-linked interventions may well have the potential to be more sustainable or scalable. Interventions designed by these system experts or ‘insiders’ may face fewer barriers to implementation and are likely to be or perceived to be in alignment with key stakeholders priorities, due to existing relationships, knowledge of the system and the ability to enact changes. Our results suggest that the manualised intervention content which can be delivered as part of the ‘Guidance and Counselling' curriculum will align with key priorities, and future research to explore whether this facilitates implementation is recommended. There may be particular challenges working within hierarchical organisations, however, such as the Church or within the wider education context, where power dynamics interplay and the intervention will need to be responsive to these. Our results suggest, for instance, that there are polarised views on the use of corporal punishment in schools from different stakeholders, including parents, headteachers, and teachers. Balancing the aims of the intervention to prevent violence from teachers within such power hierarchies within schools, where senior staff may hold positive views towards use of violent discipline, may be more difficult to navigate for intervention actors that are embedded within schools compared to ‘outsiders’.

Sustainability, defined as a continuation of “program components and activities for the continued achievement of desirable program and population outcomes” [43] (p. 2060), is often the final step in intervention development, after design and evaluation, but integrating sustainability within the initial design process is crucial [44–46]. Several frameworks exist to guide the evaluation of intervention implementation which refer to concepts of scale implementation and sustained delivery [47, 48], but neither these frameworks nor empirical evidence provides consideration of how design stage factors may influence scalability and sustainability. Within the Safe Schools Programme, design stage factors including community engagement with the Church, school leadership training, and school wide safeguarding policy implementation may be key enablers to achieving sustainability, as indicated by prior research [49, 50]. Many of these elements are responsive to the context explored in the research findings, including the need for religion to play a role in Safeguarding [4].

Scale-up involves efforts to expand the impact of successful public health interventions or innovations to benefit more people and create lasting policies and programmes [51]. Within broader implementation research, evidence on scaling up child-focused interventions is small but growing, with recent research on scaling social norms interventions [52], early childhood development [53], and parenting interventions [54]. Complex interventions may prove difficult to simplify for scale-up without altering the effective aspects of the intervention [55]. For example, interventions that rely on specialist change agents can be challenging to adapt at scale due to limited expertise or geographical constraints. Interventions initiated and designed to be embedded within existing education, religious, or child protection systems– such as the Safe Schools Programme - may provide a solution to these challenges. Our results suggest that within the Safe Schools Programme, there are several opportunities for embedding such interventions at a school level due to ZCBC’s role in school management including: new safeguarding staff will be appointed by ZCBC, teachers will act as change agents and will be trained by senior internal ZCBC staff, and existing lesson structures can be utilised for the intervention curriculum. In contrast, we found that several teachers adapted the manual content when delivering the curriculum to students, and therefore regular monitoring to ensuring messaging is not diffuse will be a potential challenge for scale up. Future research could explore the importance of these factors for successful scale-up, alongside the capacity of the intervention actors to deliver a large-scale intervention embedded within schools and the role of different school and religious actors in efforts to scale-up. Previous research suggests that the social influence of key actors is important [56] and documenting this within and outside schools in church and government ministry structures in future research would be valuable. Designing referral structures within schools to align with the wider child protection structures could also be key to future scale-up and achieving sustainability. Prior research has found the wider policy context may be crucial to sustainability of school-based interventions [50] and given the changing child protection landscape discussed above, this presents an opportunity to position the intervention alongside these changes. Given these factors, structuring the intervention to align with these existing systems could be important to achieving scale-up and sustainability.

### Strengths and limitations

There are several strengths and limitations in our approach. The three ToC workshops and subsequent ToC discussion, along with the qualitative research studies, was a lengthy process, allowing ample time for reflection and discussion between the research partners and the implementation team. This also meant that throughout the intervention design process, the content was refined in response to contextually-informed research and stakeholder discussions. This process has led to a stronger intervention with the potential for sustainability. The research benefitted from an international research team. The research, however, was limited by small sample sizes due to the challenges of COVID-19. Due to subsequent school closures, we delayed interviews with school children until the next phase of our data collection. This meant that saturation was not met and there are possible additional themes that may have been identified with a larger sample size. In addition, the research was limited to a few areas of Zimbabwe which may be distinct and not representative of the wider school context. Due to funding constraints, we were unable to recontact participants to discuss the themes that emerged during the qualitative analysis. The qualitative research alongside the intervention pilot was conducted at a single time point.

## Conclusion

Through an ongoing process of reflection and research, the Safe Schools Programme has iterated from its original design. The Safe Schools Programme is one of the first Catholic Church-led interventions targeting the reduction of violence in schools that is led by non-specialist intervention actors. The intervention will be scaled across Catholic schools and therefore has the exciting potential to provide informative learning on how violence prevention interventions designed by 'insiders' and embedded into existing school and religious structures at the design stage can be scaled successfully. Future research on embedding violence prevention within systems to achieve sustainability and scale-up is recommended.

## Data Availability

All relevant data generated or analysed during this study are included in this published article and its Supporting Information files.

## Abbreviations

NGO: Non-governmental Organisation
ToC: Theory of Change
SDGs: Sustainable Development Goals
MoPSE: Ministry of Primary and Secondary Education
ZCBC: Zimbabwe Catholic Bishops Conference
MoPSLSW: Ministry of Public Service, Labour and Social Welfare
MRC: Medical Research Council
